# The cAMP effector EPAC activates Elk1 transcription factor in prostate smooth muscle, and is a minor regulator of α1-adrenergic contraction

**DOI:** 10.1186/1423-0127-20-46

**Published:** 2013-07-02

**Authors:** Martin Hennenberg, Frank Strittmatter, Henning Schmetkamp, Beata Rutz, Sebastian Walther, Christian G Stief, Christian Gratzke

**Affiliations:** 1 Department of Urology, Ludwig-Maximilians University, Munich, Germany

**Keywords:** α1-adrenoceptor, Smooth muscle contraction, Smooth muscle relaxation, Cyclic adenosin-3^′^,5^′^-monophosphate, EPAC, Elk1, Benign prostate hyperplasia (BPH), Benign prostate obstruction (BPO), Bladder outlet obstruction (BOO), Lower urinary tract symptoms (LUTS)

## Abstract

**Background:**

Prostate smooth muscle tone is regulated by α1-adrenoceptor-induced contraction and cAMP-mediated relaxation. EPAC is an effector of cAMP, being involved in smooth muscle relaxation and cell cycle control outside the lower urinary tract. Here, we investigated the expression and function of EPAC in human prostate tissues from patients undergoing radical prostatectomy.

**Results:**

mRNA and protein expression of EPAC was detected in all prostate tissues by RT-PCR and Western blot analysis. Immunoreactivity was observed in stromal cells, and colocalized with immunofluorescence for α-smooth muscle actin and calponin. Under normal conditions, noradrenaline- or phenylephrine-induced contraction of prostate strips in the organ bath was not affected by the EPAC activator pCPT (SP-8-pCPT-2^′^-O-Me-cAMPS.NA) (30 μM). However, when the cyclooxygenase inhibitor indomethacin (50 μM) was added, EPAC activators pCPT and OME (8-CPT-2^′^-O-Me-cAMP.Na) (30 μM) significantly reduced contractions by low concentrations of phenylephrine. These effects were not observed on noradrenaline-induced contraction. OME and pCPT caused phosphorylation of the transcription factor Elk1 in prostate tissues. Elk1 activation was confirmed by EMSA (electrophoretic mobility shift assay), where OME and pCPT incresed Elk1 binding to a specific DNA probe.

**Conclusions:**

EPAC activation may reduce α1-adrenergic prostate contraction in the human prostate, although this effect is masked by cyclooxygenases and β-adrenoceptors. A main EPAC function in the human prostate may be the regulation of the transcription factor Elk1.

## Background

Cyclic adenosine-3′,5′-monophosphate (cAMP) mediates smooth muscle relaxation in the prostate and other organs [1-3]. Prostate smooth muscle tone depends on β-adrenoceptor/cAMP-mediated relaxation and α1-adrenoceptor-induced contraction, besides other mechanisms [4-6]. In patients with benign prostate syndrom (BPS), enhanced prostate smooth muscle tone and prostate enlargement may cause lower urinary tract symptoms (LUTS) [7-9]. Prostate tone and growth may be targeted by treatment with α1-blockers and 5α-reductase inhibitors, which are important therapeutic options for medical treatment of LUTS in patients with BPS [10]. Due to the high incidence of BPS and LUTS, together with the importance of smooth muscle contraction for therapy, the function of the adrenergic system in the prostate and its pharmacologic modulation are of high interest [10].

cAMP is produced by adenylyl cyclases, upon stimulation of β-adrenoceptors or with cyclooxygenase-derived prostaglandins [11,12]. After its formation, cAMP activates protein kinase A (PKA) to induce relaxation, but causes parallel interventions into gene transcription [1]. Alternatively to PKA activation, cAMP may activate “exchange proteins directly activated by cAMP” (EPAC) [11,13]. EPACs represent a group of cAMP effectors, which mediate cAMP effects independently from PKA [11,13]. Both isoforms, EPAC1 and EPAC2 were recently described from different cell types and organs, including smooth muscle outside the lower urinary tract [11,13-16]. Motoric effects of EPACs in smooth muscle have been regarded just recently, using EPAC-specific activators [16]. Activation of EPACs by these activators caused relaxation of airway smooth muscle [16]. Besides these motoric effects, EPAC activation by cAMP or specific activators results in activation of different transcription factors [17], which is involved in EPAC-mediated regulation of cell cycle [11,13,17]. Previous studies suggested that Elk1 may be activated by cAMP-dependent mechanisms in different organs and cell types [18-23]. Of note, EPAC and cAMP-dependent Elk1 activation are involved in hyperplastic alterations outside the lower urinary tract [13,18,19,24,25]. Although hyperlasia is of utmost importance for BPS, EPAC-driven Elk1 activation has not been investigated in the prostate.

Prostate smooth muscle tone is balanced by cAMP-mediated relaxation and α1-adrenergic contraction, while prostate growth requires the activation of transcription factors [24]. Prostate growth depends on the concerted interaction between growth factors, hormones and G protein-coupled receptors, although little is known about their intracellular mediators [26,27]. Prostate growth and contraction were regarded as separate phenomenons for decades (“dynamic” and “static” component) [9]. However, it has been recently postulated that both components may be coupled to each other, although detailed mechanisms still remain an enigma [4,8,28].

In the lower urinary tract, expression and function of EPACs has not been investigated to date. Here, we investigated the expression of EPAC1 and EPAC2 in the human prostate, and studied the effects of EPAC activators on adrenergic prostate contraction and on the transcription factor Elk1.

## Methods

### Human prostate tissue

Human prostate tissues were obtained from patients undergoing radical prostatectomy for prostate cancer, but without previous transurethral resection of the prostate (TURP) (n=61). The research was carried out in accordance with the Declaration of Helsinki of the World Medical Association, and has been approved by the ethics committee of the Ludwig-Maximilians University, Munich, Germany. Informed consent was obtained from each patient. All samples were taken from the periurethral zone, and analyzed anonymously. These tissue samples did not exhibit histological signs of neoplasia, cancer, or inflammation. Most prostate tumors are located to the peripheral zone [29,30].

### Sampling and in vitro stimulation

For analysis of EPAC expression, samples of prostate tissue were shock frozen in liquid nitrogen directly after prostatectomy and pathological examination. For myographic measurements of contractility, tissues were handled as described below. For in vitro stimulation with EPAC activators, prostate tissue specimens were prepared as small strips (2–3 mm × 1 mm) and allocated to three dishes of a 6-well plate containing Custodiol solution. During the experiments, plates were kept at 37°C under continous shaking. For stimulation with EPAC activators, 10 mM stock solutions were added in the required intervals and volumes to obtain a final concentration of 30 μM pCPT or OME, while another sample remained unstimulated. After 2 h, stimulated and unstimulated samples were simultaneously shock frozen in liquid nitrogen. Samples were stored at −80°C until Western blot analysis was performed.

### Quantitative RT-PCR

RNA from frozen prostate tissues was isolated using the RNeasy Mini kit (Qiagen, Hilden, Germany). For isolation, 30 mg of tissue was homogenized using the FastPrep^®^-24 system with matrix A (MP Biomedicals, Illkirch, France). RNA concentrations were measured spectrophotometrically. Reverse transcription to cDNA was performed with 1 μg of isolated RNA using the Reverse Transcription System (Promega, Madison, WI, USA). RT-PCR for EPAC1 and EPAC 2 was performed with a Roche Light Cycler (Roche, Basel, Switzerland) using primers provided by SA Biosciences (Frederick, MD, USA) as ready-to-use mixes, based on the RefSeq Accession numbers NM_006105 for EPAC1 (human RAPGEF3), and NM_007023 for EPAC2 (human RAPGEF4). PCR reactions were performed in a volume of 25 μl containing 5 μl LightCycler^®^ FastStart DNA Master^Plus^ SYBR Green I (Roche, Basel, Switzerland), 1 μl template, 1 μl primer, and 18 μl water. Denaturation was performed for 10 min at 95°C, and amplification with 45 cycles of 15 sec at 95°C followed by 60 sec at 60°C. The specificity of primers and amplification was demonstrated by subsequent analysis of melting points, which revealed single peaks for each target. The results were expressed as the number of cycles (Ct), at which the fluorescence signal exceeded a defined treshold.

### Western blot analysis

Frozen prostate tissues were homogenized in a buffer containing 25 mM Tris/HCl, 10 μM phenylmethanesulfonyl fluoride, 1 mM benzamidine, and 10 μg/ml leupeptine hemisulfate, using the FastPrep^®^-24 system with matrix A (MP Biomedicals, Illkirch, France). After brief centrifugation, supernatants were assayed for protein concentration using the Dc-Assay kit (Biorad, Munich, Germany) and boiled for 10 min with sodium dodecyl sulfate (SDS) sample buffer (Roth, Karlsruhe, Germany). Western blot analyses of samples were performed as previously described [31]. For detection, mouse anti EPAC1 (5D3) antibody, mouse anti EPAC2 (5B1) antibody (both from New England Biolabs, Ipswich, MA, USA), mouse anti phospho-Elk1 (serine 383) antibody (B-4), mouse anti Elk1 antibody (3H6D12), mouse anti pan-cytokeratin antibody (C11), mouse anti prostate specific antigen (PSA) antibody (A67-B/E3), or mouse anti β-actin antibody (all from Santa Cruz Biotechnology, Santa Cruz, CA, USA) were used. Blots were developed with enhanced chemiluminescence (ECL) using ECL Hyperfilm (GE Healthcare, Freiburg, Germany). Intensities of the resulting bands were quantified using Image J (NIH, Bethesda, Maryland, USA). In stimulation experiments with EPAC activators, samples without and with activator were compared on one blot, and subjected to semiquantitative quantification. For quantification, samples without activator were set to 100%, and data of stimulated samples from the same prostate were expressed as % of these unstimulated samples.

### Immunohistochemistry

Sections (6–8 μm) from frozen tissues were stained by an indirect immunoperoxidase technique, as previously described [31]. For detection of EPAC1 and EPAC2, mouse anti EPAC1 antibody (5D3) or EPAC2 antibody (5B1) (New England Biolabs, Ipswich, MA, USA) were used in dilutions of 1:200. Biotinylated secondary horse anti mouse antibody (Vector Laboratories, Burlingame, CA, USA) and avidin-biotin-peroxidase complex (Vector Laboratories, Burlingame, CA, USA) were sequentially applied for 30 minutes each. Staining was performed with the AEC peroxidase substrate kit (Vector Laboratories, Burlingame, CA, USA). Finally, all sections were counterstained with hemalaun. Control stainings without primary antibodies did not yield any immunoreactivity.

### Immunofluorescence

Human prostate specimens, embedded in optimal cutting temperature compound, were snap-frozen in liquid nitrogen and kept at −80°C. Sections (8 μm) were cut in a cryostat and collected on microscope slides (Superfrost^®^). Fluorescence stainings were performed as previously described [31], using the following primary antibodies (diluted 1:50): mouse anti EPAC1 (5D3), mouse anti EPAC2 (5B1) (both from New England Biolabs, Ipswich, MA, USA), rabbit anti EPAC1 (EPR1672) (Thermo Scientific, Kalamazoo, MI, USA), mouse anti Elk1 (3H6D12) (Santa Cruz Biotechnology, Santa Cruz, CA, USA), rabbit anti α-smooth muscle actin (αSMA) (RB-9010-P) (Thermo Scientific, Kalamazoo, MI, USA), and rabbit anti calponin (FL-297) (Santa Cruz Biotechnology, Santa Cruz, CA, USA). Binding sites were visualized using Cy3 and Cy5 conjugated secondary antibodies (goat anti mouse, AP124C, Millipore, Billerica, MA, USA, 1:1000; goat anti rabbit, 1:1000, ab6564, Abcam, Cambridge, UK). Nuclei were counterstained with DAPI (1:50,000, D1306, Invitrogen, Camarillo, CA, USA) during incubation with the secondary antibody. Immunolabelled sections were analysed using a laser scanning microscope (Leica SP2, Wetzlar, Germany). Control stainings without primary antibodies did not yield any signals.

### Tension measurements

Prostate strips (6×3×3 mm) were mounted in 5 ml aerated (95% O_2_ and 5% CO_2_) tissue baths (Danish Myotechnology, Aarhus, Denmark), containing Krebs-Henseleit solution (37°C, pH 7.4). Preparations were stretched to 0.5 g and left to equilibrate for 45 min to attain a stable resting tone. After the equilibration period, maximum contraction induced by 80 mM KCl was assessed. Subsequently, chambers were washed three times with Krebs-Henseleit solution for a total of 30 min. Cumulative concentraction response curves for noradrenaline or for the α1-adrenergic agonist phenylephrine were created before and after addition of EPAC activators (30 μM pCPT, or 30 μM OME). In experiments including indomethacin, this was added already during the equilibration period, and applied throughout the entire experiment.

### EMSA

Activation of Elk1 was investigated by non-radioactive electrophoretic mobility shift assay (EMSA). In this assay, the binding of Elk1 to a biotin-labelled, Elk1-specific DNA probe is determined. Assays were performed using a commercially available kit (Affymetrix, Santa Clara, CA, USA) according to the manufacturer’s instruction. In brief, prostate tissues were homogenized as described for Western blot analysis, but not boiled with sample buffer. After protein determination, 20 μg of protein were incubated with biotin-labelled DNA probe with the sequence 5′TTTGCAAAATGCAGGAATTGTTTTCACAGT′3. After incubation, samples were subjected to electrophoresis in native, non-denaturating acrylamide gels (6%), and subsequently blotted on nylon membranes, where detection for biotin was performed with peroxidase-coupled streptavidin in combination with ECL. Intensities of the resulting bands were quantified using Image J (NIH, Bethesda, Maryland, USA). Experimental conditions were approved by preparation of a negative control using an unlabelled probe provided by the manufacturer. This cold probe was added to a sample besides the labelled probe, resulting in competition and disappearence of bands.

### Drugs and solutions

8-(4-chlorophenylthio)-2′-O-methyladenosine-3′,5′-cyclic monophosphate sodium salt (“OME”) (Axxora, San Diego, CA, USA) and 8-(4-chlorophenylthio)-2′-O-methyladenosine-3′,5′-cyclic monophosphorothiorate SP-isomer (“pCPT”) (BioLog, Bremen, Germany) are specific, isoform-unselective activators of EPAC [32,33]. Both were dissolved in water and kept as 10 mM stock solutions at −20°C until use. Aqueous stock solutions for noradrenaline and of the α1-adrenoceptor agonist phenylephrine (Sigma, St. Louis, MO, USA) (10 mM) were freshly prepared for each experiment.

### Statistical analysis

Data are presented as means ± standard error of the mean (SEM) with the indicated number (n) of experiments. Two-tailed student *t* test was used for paired or unpaired observations. *P* values <0.05 were considered statistically significant.

## Results

### Quantitative RT-PCR

**Figure 1:**
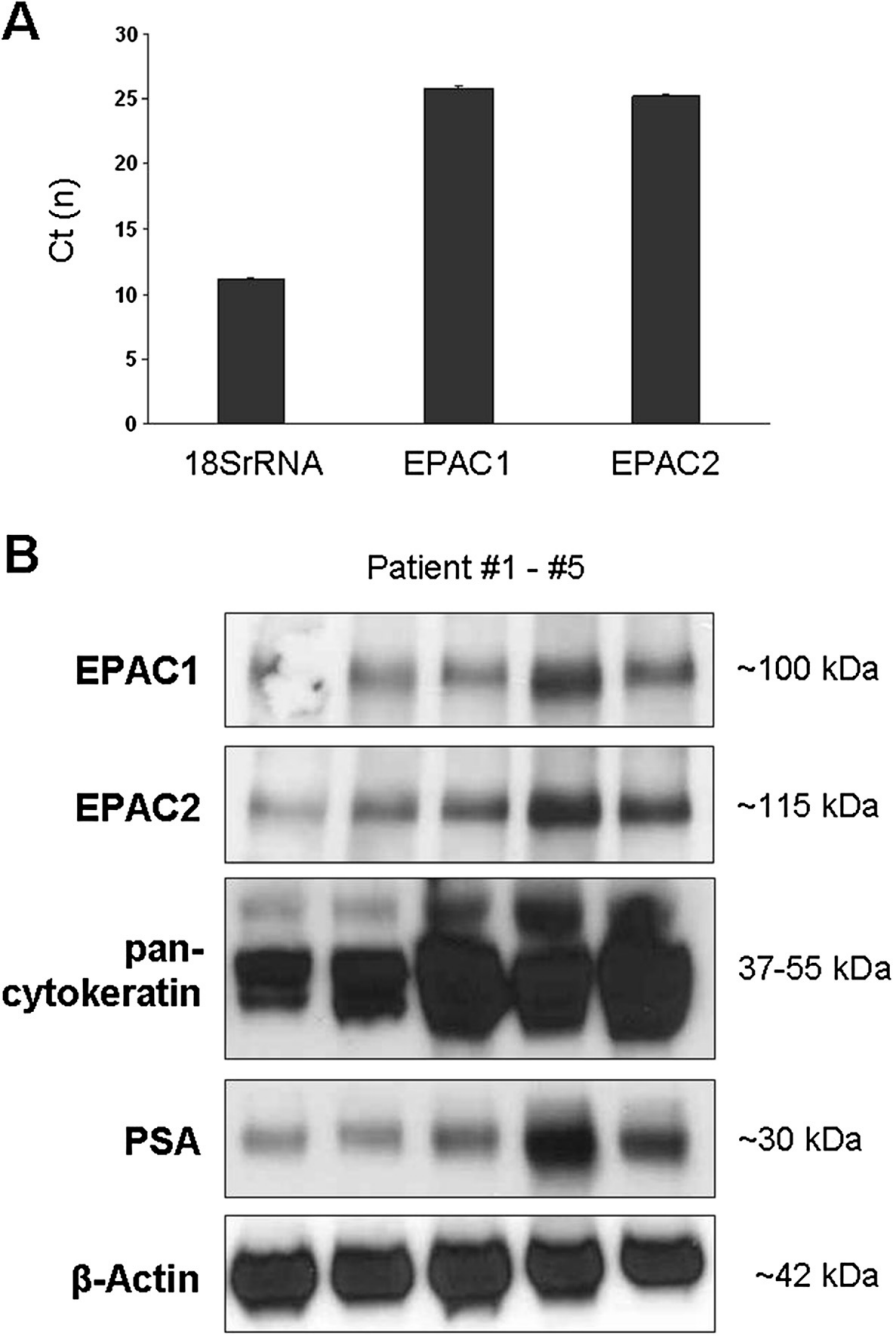
**EPAC expression in the human prostate.** mRNA **(A)** and protein **(B)** expression of EPAC 1 and EPAC2 in human prostate tissues. In **(A)**, mRNA expression was investigated by quantitative RT-PCR in prostate tissues from n=5 patients (data are means ± SEM). In **(B)**, protein expression of EPAC1, EPAC2, pan-cytokeratin, PSA, and β-actin was investigated by Western blot analyses in prostate tissues from n=5 patients.

Expression of EPAC1 and EPAC2 mRNA was detected in prostate samples from all investigated patients (n=5). Average Ct was 26 ± 0.3 for EPAC1, and 25 ± 0.2 for EPAC2, while the housekeeping gene 18SrRNA was detectable with an average Ct of 11 ± 0.2 (Figure 1A).

### Western blot analysis of EPAC expression

Western blot analysis using isoform-specific EPAC antibodies demonstrated variable protein expression of EPAC1 and EPAC2 in prostate tissues of all investigated patients (n=5). Detected bands matched the expected sizes for both isoforms (96 kDa for EPAC1, 115 kDa for EPAC2) (Figure 1B). The intensity of bands for EPAC1 and EPAC varied between different patients (Figure 1B). The content of epithelial markers, pan-cytokeratin (37–55 kDa) and PSA (30 kDa) varied between prostates of different patients (Figure 1B). The content of β-actin was similar in samples of different patients (Figure 1B).

### Double fluorescence staining

**Figure 2:**
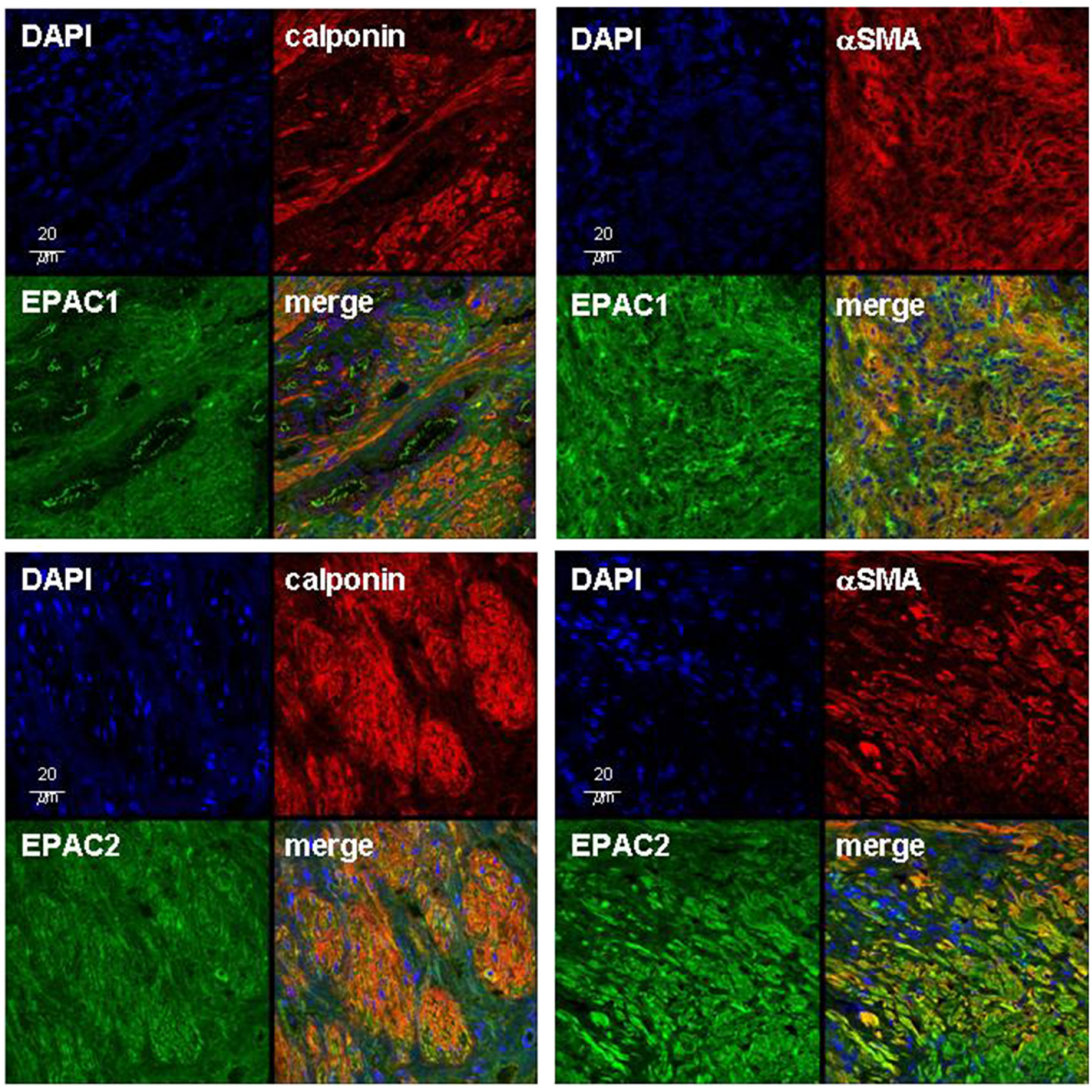
**Double fluorescence stainings of human prostate sections for EPAC1 or EPAC2, and α-smooth muscle actin (αSMA) or calponin.** Stainings were performed using isoform-specific EPAC antibodies. Colocalization of EPAC with αSMA- or calponin-positive cells resulted in yellow color in merged pictures after overlay. Shown are representative stainings from experiments with tissues from n=5 patients with similar results.

Fluorescence staining of prostate sections resulted in immunoreactivity for EPAC1 and EPAC2, and for the smooth muscle markers α-smooth muscle actin (αSMA) and calponin in prostate tissues from all investigated patients (n=5) (Figure 2). Virtually all αSMA- and calponin-positive cells were immunoreactive for EPAC1 and EPAC2 (Figure 2). This colocalization was indicated by yellow color in merged pictures after overlay. No immunoreactivities were observed in control experiments, where the primary antibodies were replaced by PBS.

**Figure 3:**
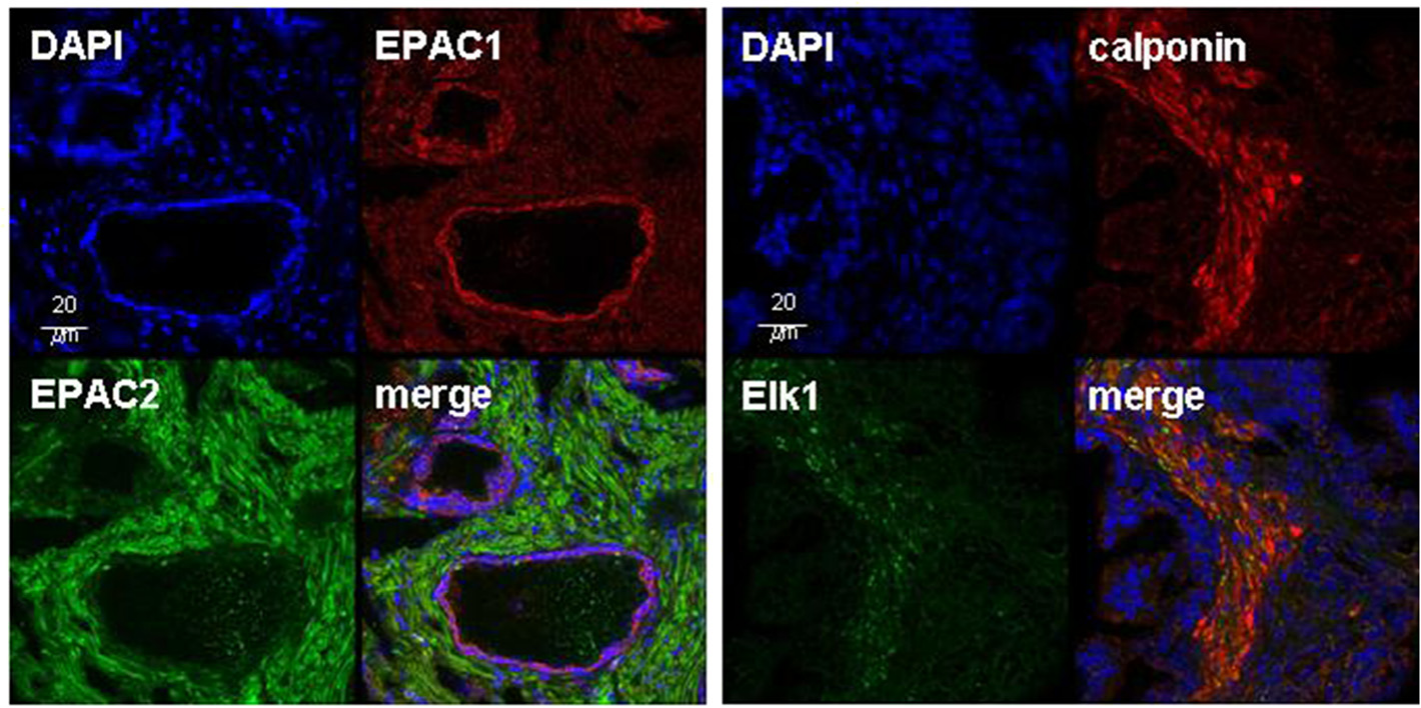
**Double fluorescence stainings of human prostate sections for EPAC1 and EPAC2 (left), or for Elk1 and calponin (right).** Colocalization resulted in yellow color in merged pictures after overlay, which is slightly visible after double labelling for Elk1 and calponin. Shown are representative stainings from experiments with tissues from n=5 patients with similar results.

After double labelling for EPAC1 and EPAC2, immunoreactivity for EPAC1 was strongest in epithelial cells, but also observed in the stroma (Figure 3). In contrast, immunoreactivity for EPAC2 was strong in the stroma, but almost absent in epithelial cells (Figure 3). Colocalization of EPAC1 and EPAC2 was not observed (Figure 3). After double labelling for Elk1 and calponin, immunoreactivity for Elk1 was observed in the stroma (Figure 3). In epithelial cells, almost no Elk1 immunoreactivity was observed (Figure 3). In merged pictures, yellow color indicating colocalization of Elk1 and calponin was weak, but detectable (Figure 3).

### Immunohistochemical staining

**Figure 4:**
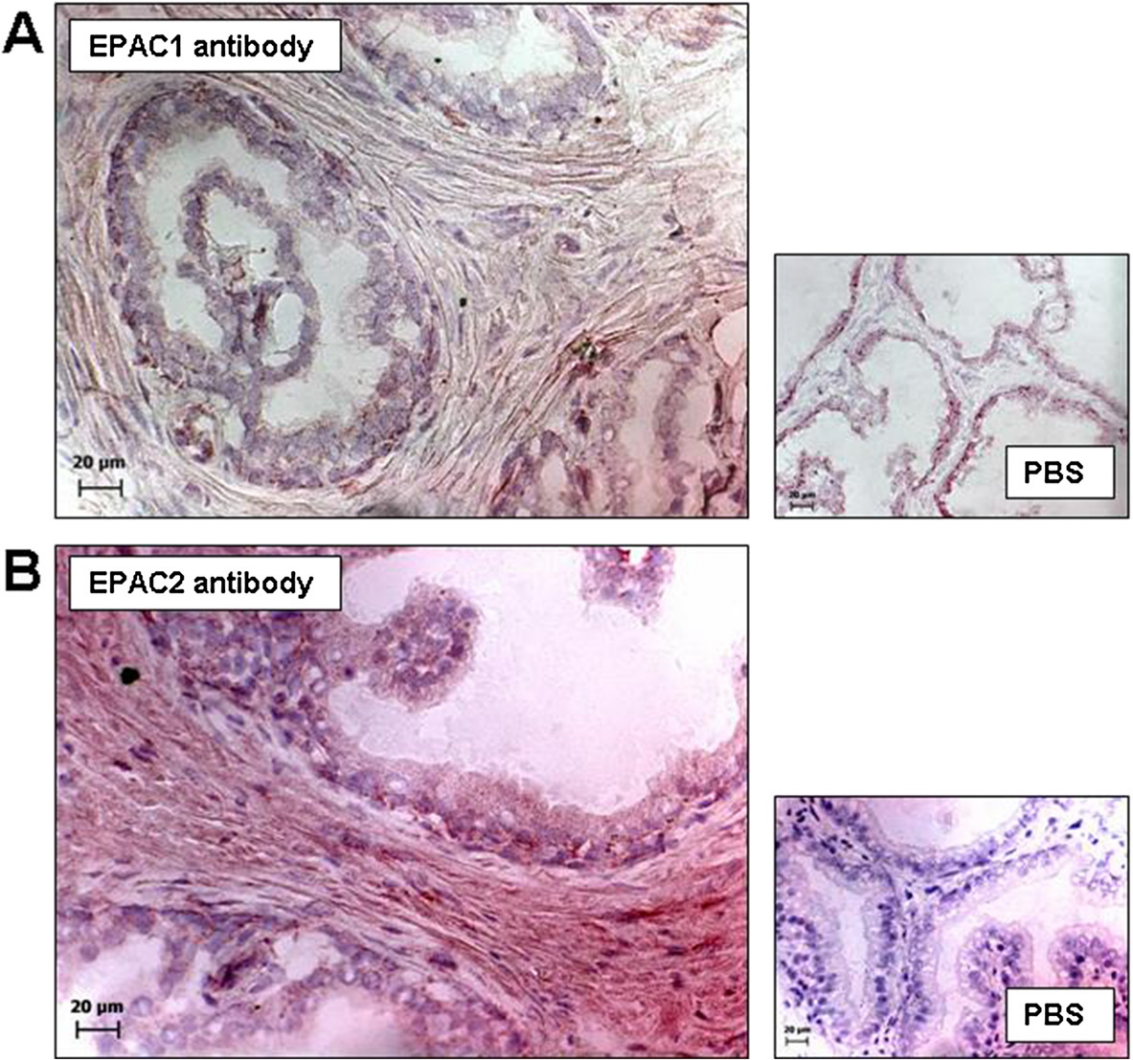
**Immunohistochemical stainings of human prostate sections with isoform-specific EPAC antibodies.** Peroxidase-based stainings were performed using an EPAC1 antibody in **(A)**, or an EPAC2 antibody in **(B)**. Immunoreactivity resulted in brown color. In control stainings, primary antibodies were replaced by phosphate-buffered saline (PBS). Shown are representative stainings from series with prostates from n= 5 patients in **(A)** and **(B)**.

Immunohistochemical staining of prostate sections using EPAC1 and EPAC2 antibodies resulted in immunoreactivites in stromal cells (n=5 patients) (Figure 4). In control experiments, where antibodies were replaced by PBS, no immunoreactivities were observed (Figure 4).

### Tension measurements

**Figure 5:**
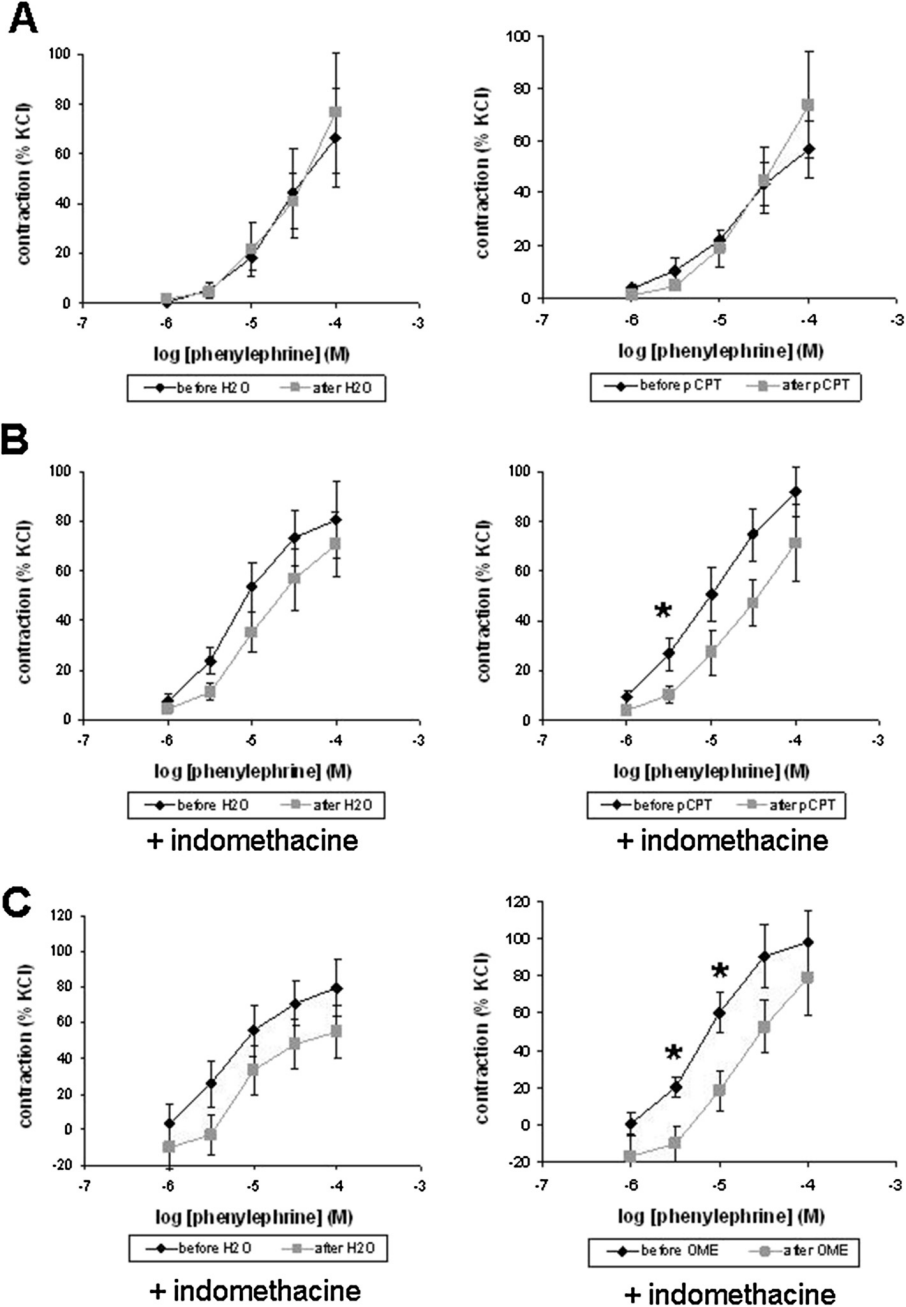
**Effects of pCPT and OME on phenylephrine-induced contraction of human prostate strips.** Phenylephrine-induced contraction was measured before and after application of pCPT, OME, or water (solvent). In **(B)** and **(C)**, the inhibitor of cyclooxyenases, indomethacin (50 μM) was added during the entire experiment. Data are means ± SEM from experiments with prostates from n=2 patients in **(A)**, n=7 patients in **(B)**, and n=8 patients in **(C)** (* p<0.05 vs. pCPT, ** p<0.02 vs. OME).

In myographic measurements, phenylephrine and noradrenaline induced concentration-dependent contractions of isolated prostate strips. Under normal conditions, pCPT (30 μM) was without effects on phenylephrine-induced contractions (Figure 5A). When the cyclooxygenase inhibitor indomethacin (50 μM) was added before construction of concentration response curves, pCPT significantly reduced contraction by 3 μM phenylephrine (p<0.05) (Figure 5B). Similarly, OME (30 μM) significantly reduced contractions by 3 μM (p<0.02) and 10 μM phenylephrine (p<0.02), when indomethacin was added (Figure 5C).

**Figure 6:**
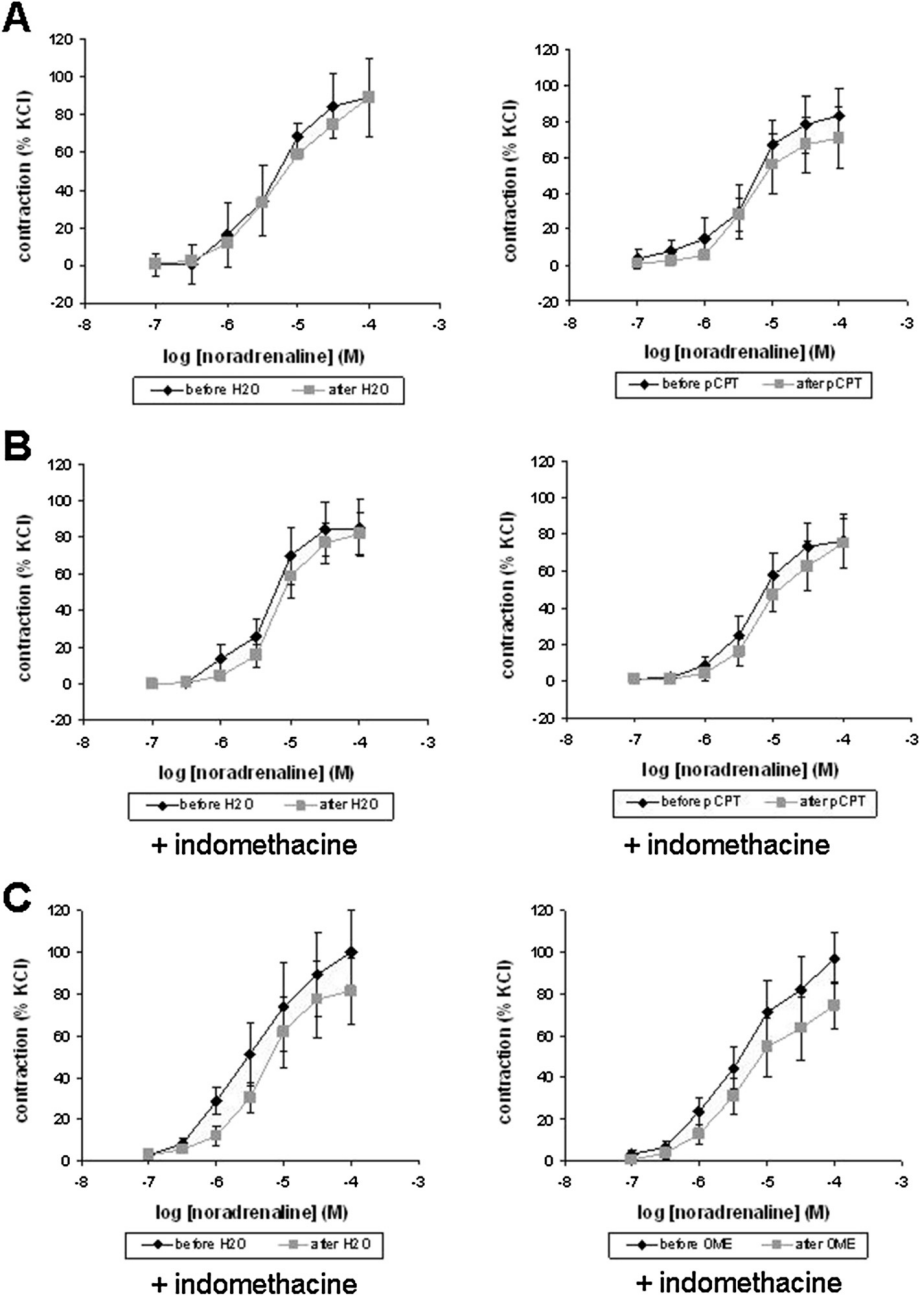
**Effects of pCPT and OME on noradrenaline-induced contraction of human prostate strips.** Noradrenaline-induced contraction was measured before and after application of pCPT, OME, or water (solvent). In **(B)** and **(C)**, the inhibitor of cyclooxyenases, indomethacin (50 μM) was added during the entire experiment. Data are means ± SEM from experiments with prostates from n=6 patients in **(A)**, n=7 patients in **(B)**, and n=5 patients in **(C)**.

In contrast, pCPT was without effects on noradrenaline-induced contractions, regardless whether indomethacin was added or not (Figure 6A,B). Similarly, OME was without effect on noradrenaline-induced contractions in the presence of indomethacin (Figure 6C).

### Western blot analysis of Elk1 phosphorylation

**Figure 7:**
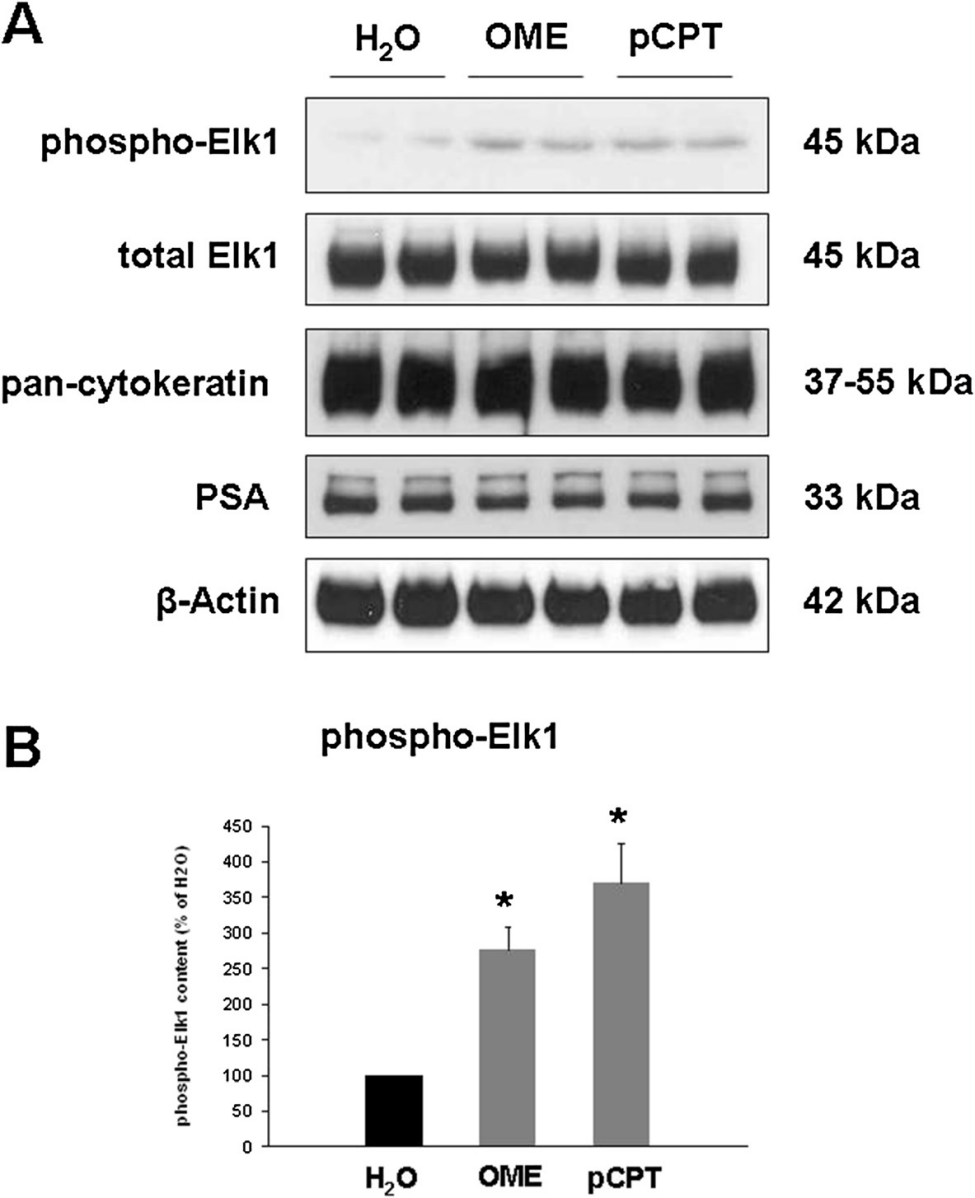
**Effects of OME and pCPT on the content of phospho-Elk1 in human prostate tissue.** Samples from one prostate were incubated for 2 h with OME (30 μM), pCPT (30 μM), or water (solvent), and subsequently analyzed for phospho-Elk1, total Elk1, pan-cytokeratin, PSA, and β-actin by Western blot analysis. Shown are representative Western blots in **(A)**, and densitometric quantification in **(B)** from experiments with prostates from n=6 (phospho-Elk) patients (* p<0.0007 vs. H_2_O).

Using a phospho-specific antibody, the effect of OME and pCPT on Elk1 phosphorylation was determined by Western blot analysis. Incubation of prostate tissues with OME or pCPT for 2 h significantly increased the phosphorylation state of Elk1. After incubation with OME (30 μM), Elk1 phosphorylation was 276 ± 33% of unstimulated controls (p<0.0004) (Figure 7). After incubation with pCPT (30 μM), Elk1 phosphorylation was 370 ± 56% of unstimulated controls (p<0.0007) (Figure 7). The content of Elk1, pan-cytokeratin, PSA, and β-actin was similar in stimulated and unstimulated samples in each experiment (Figure 7).

### EMSA

**Figure 8:**
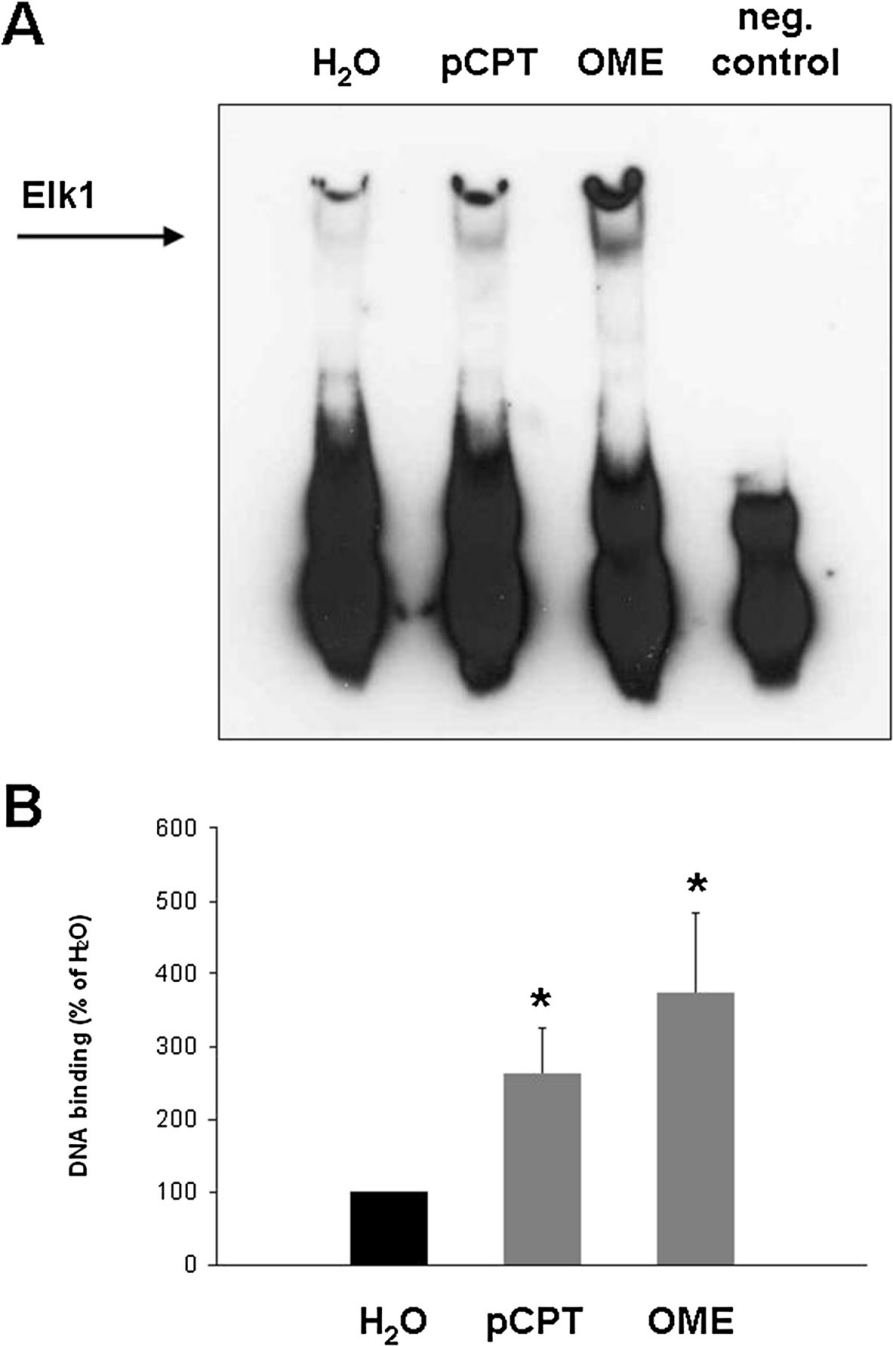
**Elk1 activation by OME and pCPT in human prostate tissue.** Samples from one prostate were incubated for 2 h with OME (30 μM), pCPT (30 μM), or water (solvent), and subsequently analyzed by an Elk1-specific EMSA. Binding of Elk1 to the specific, biotin-labelled DNA probe causes a shift in migration during electrophoresis, as indicated in **(A)**. Experimental conditions were approved by preparation of a negative control using an unlabelled probe, which was added to a sample besides the labelled probe, resulting in competition and disappearence of bands. Shown is a representative experiment in **(A)**, and densitometric quantification in **(B)** from experiments with prostates from n=5 patients (* p<0.04 vs. H_2_O).

Using an electrophoretic mobility shift assay (EMSA), we investigated Elk1 activation by EPAC activators. In this assay, the binding of Elk1 to the DNA sequence 5′TTTGCAAAATGCAGGAATTGTTTTCACAGT′3 is assessed. Incubation of prostate tissues (n=5 patients) with pCPT or OME (30 μM, 2 h) resulted in binding of Elk1 to this sequence (Figure 8). DNA binding after incubation with pCPT was 264 ± 62% of the binding in unstimulated samples (p<0.04). Similarly, DNA binding after incubation with OME was 375 ± 110% of the binding in unstimulated samples (p<0.04).

## Discussion

In the prostate and other organs, cyclic adenosine-3′,5′-monophosphate (cAMP) is a second messenger mediating smooth muscle relaxation [1]. In addition to its role for smooth muscle tone, cAMP is involved in non-motoric functions, including regulation of gene transcription or cell cycle in many cell types and organs [17,24,25]. cAMP-dependent effects may be mediated either by PKA, or by EPAC [11,13]. By PKA and EPAC, cAMP may be assorted to different intracellular compartments, and consequently to divergent cellular functions [11,13]. In smooth muscle outside the lower urinary tract, cAMP-dependent EPAC activation mediates relaxation and regulates cell cycle, besides its involvement in other functions [14-16]. Smooth muscle tone and growth are important factors contributing to the pathophysiology and therapy of LUTS in patients with BPS [7-10]. To the best of knowledge, the expression and function of EPAC in the prostate has not been investigated to date. Here, we studied EPAC expression and EPAC functions in human prostate smooth muscle, using EPAC-specific activators.

Using RT-PCR, Western blot analysis, and immunohistochemistry, we observed expression of EPAC1 and EPAC2 in prostate samples from all investigated patients. In Western blot analysis, EPAC expression levels varied together with the epithelial markers, PSA and pan-cytokeratin between prostates of different patients. Despite these variations, EPAC was detectable in all samples, indicating that a constitutive expression exists. Nevertheless, our analyses demonstrate that EPAC expression underlies regulation. The different content of epithelial markers may reflect different degrees of prostate hyperplasia. In fact, almost all patients undergoing radical prostatectomy show hyperplastic prostates, although to different extent [34,35]. Therefore, we assume that our findings reflect the situation in hyperplastic tissue, where the expression level of EPAC may vary with the degree of hyperplasia. A comparison to non-hyperplastic tissues was not possible, as these tissues are not available. The aim of our present study was to demonstrate a new principle of EPAC signaling in non-malignant prostate tissue, independent of pathophysiological context. Immunoreactivity for EPAC1 and EPAC2 was located to stromal cells. To confirm that these cells are smooth muscle cells, we performed double immunofluorescence stainings of prostate sections. Indeed, immunoreactivity for both EPAC isoforms colocalized with αSMA, which is a common marker for smooth muscle cells.

Recently, different cell-permeable EPAC activators have been developed, which are indispensable tools for investigations of EPAC functions [32,33]. These activators, are analogs of cAMP, which do not activate PKA, but are resistant to hydrolysis by phosphodiesterases [32,33]. Although OME and pCPT are specific activators of EPAC, they do not discriminate between EPAC1 and EPAC2. In our organ bath experiments, activation of EPAC caused inhibition by low concentrations of phenylephrine, when cyclooxygenase activity was blocked by indomethacin. In experiments, where indomethacin was omitted or contraction was induced by noradrenaline, EPAC activation was without effects on contraction. In contrast to noradrenaline, which activates α- and β-adrenoceptors, phenylephrine selectively activates α1-adrenoceptors. Of note, these effects were confirmed using two different EPAC activators, OME and pCPT. In conclusion, a contribution of EPAC to prostate smooth muscle tone may exist, althouth to minor extent. Cyclooxygenases and noradrenaline-induced β-adrenoceptor activation cause cAMP production [11,12]. Under physiologic conditions, this may raise EPAC activity to a level, where further EPAC activation by OME or pCPT is in ineffective on prostate smooth muscle tone. When this background of cAMP was deleted in our experiments (by indomethacin and selective activation of α1-adrenoceptors), the effect of EPAC activators on contraction became visible.

Relaxation in response to EPAC activators has been recently described from airway smooth muscle, where EPAC-mediated relaxation may exceed the effects in the prostate [16]. We assume that any difference to our study may be either explained by the divergent, organ-specific contractile systems in both organs, or by a tissue-specific equipment with different molecular EPAC effectors. Whether EPAC has a role in other smooth muscle types of the lower urinary tract, in particular within the bladder, may be subject of further studies.

Regulation of gene transcription by cAMP has been known since decades [17,36]. By interventions into transcriptional activity, cAMP is involved in various central functions, including cellular growth, differentiation and regulation of cell cycle [32,36]. In fact, different transcription factors were identified, which may be activated by cAMP and EPAC [17]. Although the focus of previous studies was on the regulation of CREB by cAMP, several studies suggested that cAMP activates Elk1 in different organs and cell types [18-23]. Therefore, we investigated whether EPAC activators may trigger Elk1 activation in the prostate. We observed that stimulation of human prostate tissues with EPAC activators results in activation of Elk1.

Elk1 is activated by a phosphorylation, resulting in binding of the factor to a specific DNA sequence within the promoter region of target genes [37,38]. Activation of Elk1 in our samples was confirmed by Western blot analysis using a phospho-specific antibody, and by EMSA, where the binding of transcription factors to a specific, biotin-labelled DNA probe is assessed. Among different functions of Elk1, Elk1-dependent proliferation, growth and differentiation have been described from smooth muscle cells and other cell types [38-41]. In the liver, cAMP-mediated Elk1 activation mediates hyperplasia of bile ducts [18,19]. In prostate cancer cells, Elk1 is involved in proliferation and tumor growth [42,43]. To the best of our knowledge, our study suggesting a link between EPAC and Elk1 activation is the first regarding Elk1 in non-malignant prostate cells, or linking EPAC to Elk1 activation in any cell type. Elk1-specific inhibitors, which may enable detailed studies on Elk1 function, have not been developed to date. We assume that EPAC uses different effectors besides Elk1 in the prostate. However, a role for Elk1 in the control of smooth muscle tone appears unlikely. Future studies may focus on the identification of Elk1 target genes in the prostate.

Non-motoric EPAC functions were studied in a panel of cell types, including smooth muscle cells outside the lower urinary tract. In airway smooth muscle cells, EPAC regulates the phenotype of smooth muscle cells, and inhibited growth factor-induced proliferation [14,15]. Apart from smooth muscle cells, the role of EPAC was studied in different cell types, with diverging results. In prostate carcinoma cells, an antiproliferative effect as well as EPAC-driven proliferation was observed [44,45]. EPAC triggers proliferation in endothelial cells, macrophages, thyroid cells, or osteoblasts, [46-49]. Indeed, the opposing character of EPAC functions, in particular with regard to cell cycle regulation already attracted attention [32]. Interestingly, EPAC functions are often associated with the same biological processes, despite opposing effects [11].

Together, EPAC-specific activators induce activation of the transcription factor Elk1 in the human prostate. In contrast, EPAC-mediated relaxation of prostate smooth muscle may be at best minor. Nevertheless, cAMP is an important mediator causing prostate smooth muscle relaxation by PKA [1]. This may suggest possible connections between smooth muscle tone and growth in the prostate. Although such links have been proposed by various investigators, little is known about their intracellular mediators [4,8,26-28]. In cardiomyocytes, EPAC activation causes hypertrophic responses, by intervention into transcription of hypertrophic genes [13,24,25]. In conclusion, a role of EPAC in prostate hyperplasia may be postulated.

## Conclusions

Our findings point to a role of EPAC in transcriptional regulation in smooth muscle cells of the human prostate. EPAC-dependent regulation of prostate smooth muscle tone may be masked by cyclooxygenases and β-adrenoceptors. Together, EPAC may represent a missing link connecting the dynamic with the static component in BPH.

## Abbreviations

BPS: Benign prostate syndrom; cAMP: Cyclic adenosin-3^′^,5^′^-monophosphate; CREB: cAMP response element-binding protein; EMSA: Electrophoretic mobility shift assay; EPAC: Exchange proteins directly activated by cAMP; LUTS: Lower urinary tract symptoms; OME: 8-CPT-2^′^-O-Me-cAMP.Na; PBS: Phosphate-buffered saline; pCPT: SP-8-pCPT-2^′^-O-Me-cAMPS.NA; PKA: Protein kinase A; PSA: Prostate-specific antigen; RT-PCR: Real time polymerase chain reaction; TURP: Transurethral resection of the prostate.

## Competing interests

The authors declare that they have no competing interests.

## Authors’ contributions

MH made substantial contributions to the conception and design, analysis and interpretation of data, drafting of the manuscript, and performed in vitro stimulation experiments. FS contributed to the acquisition of prostate tissues (performed radical prostatectomy), was involved in organ bath experiments, and critically revised the manuscript for important intellectual content. HS performed organ bath experiments, was involved in analyses of resulting data, and critically revised the manuscript for important intellectual content. BR performed molecular analyses of tissues (stainings, Wester blotting, EMSA), contributed to analysis of data, and critically revised the manuscript for important intellectual content. SW contributed to the acquisition of prostate tissues (performed radical prostatectomy), was involved in organ bath experiments, and critically revised the manuscript for important intellectual content. CGS contributed to the acquisition of prostate tissues (performed radical prostatectomy), to the conception and design, and critically revised the manuscript for important intellectual content. CG contributed to the acquisition of prostate tissues (performed radical prostatectomy), to the conception and design, and critically revised the manuscript for important intellectual content. All authors have given final approval of the manuscript.
